# NeuroD1-based *in situ* neural regeneration for the treatment of radiation-induced brain injury

**DOI:** 10.4103/NRR.NRR-D-24-01067

**Published:** 2025-01-29

**Authors:** Xudong Yan, Ke Zhong, Meijuan Zhou, Jiao Chen, Yajie Sun, Yamei Tang, Gong Chen, Yongteng Xu

**Affiliations:** 1 Guangdong-Hong Kong-Macau Institute of CNS Regeneration, Jinan University, Guangzhou, Guangdong Province, China; 2 Department of Neurology, Sun Yat-Sen Memorial Hospital, Sun Yat-Sen University, Guangzhou, Guangdong Province, China; 3 Brain Research Center, Sun Yat-sen Memorial Hospital, Sun Yat-sen University, Guangzhou, Guangdong Province, China; 4 Department of Pharmacy, Sun Yat-Sen Memorial Hospital, Sun Yat-Sen University, Guangzhou, Guangdong Province, China; 5 Department of Radiation Medicine, Guangdong Provincial Key Laboratory of Tropical Disease Research, School of Public Health, Southern Medical University, Guangzhou, Guangdong Province, China

**Keywords:** angiogenesis, bulk RNA sequencing, hemorrhagic stroke, *in situ* neural regeneration, magnetic resonance imaging, NeuroD1, neuroinflammation, radiation-induced brain injury, reactive astrocytes, transdifferentiation

## Abstract

Radiation-induced brain injury remains one of the most severe complications of radiotherapy for head and neck tumors, with limited options for prevention and treatment. *In situ* neural regeneration technology has demonstrated promising therapeutic effects in various neurodegenerative and neurotrauma conditions. In this study, we overexpressed the neural transcription factor NeuroD1 using *in situ* neural regeneration technology in a radiation-induced brain injury mouse model. This approach converted reactive astrocytes into neurons, increased neuronal density, protected endogenous neurons, decreased microglial activation, reduced peripheral CD8^+^ T cell infiltration, and diminished angiogenesis in the injured area, leading to a significant reduction in lesion volume. Additionally, we explored the potential mechanisms of NeuroD1 *in situ* neural regeneration technology through bulk RNA sequencing, which showed an upregulation of neurogenesis-related genes and a downregulation of immune response–related and angiogenesis-related genes. Furthermore, our findings suggested that NeuroD1 *in situ* neural regeneration technology converted reactive astrocytes into neurons and reduced microglial activation in a thalamic hemorrhagic stroke mouse model. In summary, this study supports NeuroD1 *in situ* neural regeneration technology as a potential therapeutic approach for treating radiation-induced brain injury and hemorrhagic stroke, and offers new insights into the therapeutic role of NeuroD1 in delayed brain injury.

## Graphical abstract

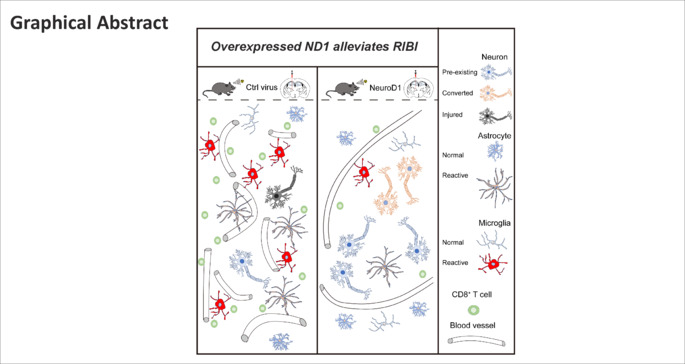

## Introduction

Radiotherapy is a well-established treatment for head and neck tumors, such as nasopharyngeal carcinoma, but it unavoidably causes damage to the surrounding healthy brain tissue (Tang et al., 2012; Chargari et al., 2019; Chen et al., 2019). The incidence of radiation-induced brain injury (RIBI) that results from radiotherapy is influenced by various factors, including the radiotherapy mode, total radiation dose, and radiation area (Ali et al., 2019). Approximately 30% of patients treated with radiotherapy for head and neck tumors suffer from RIBI (Cheng et al., 2023). Patients with RIBI may experience early clinical symptoms such as headache and lethargy. In advanced stages, they may develop cognitive dysfunction and necrosis of brain tissue, ultimately leading to death (Tang et al., 2012; Shen et al., 2016; Shi et al., 2023a). Magnetic resonance imaging (MRI) is widely used for diagnosing RIBI because it provides detailed anatomical and spatial information about the brain injury (Rogers et al., 2011; Ngen et al., 2016; Mayo et al., 2024). Currently, the response rate to therapeutic options for RIBI, including corticosteroids and the vascular endothelial growth factor monoclonal antibody bevacizumab, is limited (Pan et al., 2022). For example, corticosteroid therapy demonstrates efficacy in only 20%–31.5% of patients and is frequently accompanied by significant side effects (Chao et al., 2013; Cheng et al., 2023). Bevacizumab therapy has shown better results than corticosteroids, with efficacy in 50%–65.5% of patients with RIBI. However, it is also limited by its short-lived efficacy and high risk of intracerebral hemorrhage (Levin et al., 2011; Khasraw et al., 2012; Xu et al., 2018; Zhuang et al., 2019; Cheng et al., 2023). Thus, novel treatment strategies for RIBI should urgently be explored.

The mechanism underlying RIBI is still largely unknown. In addition to neuronal injury directed caused by radiation, the microenvironment of the radiation area is vital for the survival of injured neurons. The microenvironment around injured neurons in the radiation area is complex, involving activated microglia, reactive gliosis, a disrupted blood–brain barrier (BBB) and infiltrated immune cells (Yang et al., 2017; Ma et al., 2023). In RIBI animal models, typical pathological features include microglia activation, astrocyte proliferation, BBB disruption, and irreversible neuronal death (Balentova and Adamkov, 2015; Ali et al., 2019; He et al., 2020; Liu et al., 2022). Astrocytes play crucial roles in maintaining the integrity and function of the neuronal network, microenvironment homeostasis, and BBB integrity under physiological conditions (Abbott et al., 2006; Verkhratsky and Nedergaard, 2018). Under pathological conditions, astrocytes proliferate and become inflammatory activation through BBB remodeling, cytokine secretion and scar formation. Astrocytes are key regulators of the metabolic and inflammatory landscape of the central nervous system and have emerged as potential therapeutic targets for a variety of disorders (Tsai et al., 2012; Magistretti and Allaman, 2018; Singh, 2022; Patani et al., 2023). Astrocyte proliferation is commonly observed in the necrotic brain area of RIBI animal models and patients, suggesting that astrocyte proliferation may contribute to the development of RIBI (Furuse et al., 2015; Vellayappan et al., 2018; Garcia et al., 2020; He et al., 2020; Shi et al., 2023a). Whether targeting astrocytes could serve as a therapeutic strategy for RIBI remains uninvestigated.

Recent advancements in *in situ* neuronal regeneration technology have shown potential for the treatment of neurodegenerative diseases and neurotrauma. This technology converts endogenous reactive astrocytes into neurons by overexpressing the transcription factor NeuroD1 (ND1) in astrocytes, which is associated with neuronal development. Studies have demonstrated promising therapeutic effects in rodent models of Alzheimer’s disease, ischemic stroke, Huntington’s disease, and temporal lobe epilepsy (Guo et al., 2014; Chen et al., 2020; Ge et al., 2020; Wu et al., 2020; Zheng et al., 2022). Compared with exogenous cell replacement therapies, such as induced pluripotent stem cells, neurons regenerated *in situ* from endogenous reactive astrocytes in the brain evade immune rejection and tumorigenesis risk, and integrate seamlessly into the native neural circuits (Li and Chen, 2016; Barker et al., 2018; Wang et al., 2021b). ND1 is a neural transcription factor closely related to neurogenesis, expressed not only during early brain development but also in small amounts in the cortex and hippocampus of adult mice (Gao et al., 2009; Kuwabara et al., 2009; Guo et al., 2014). Studies on *in situ* neuronal regeneration have also showed that ND1 overexpression not only increases neuronal regeneration (Brulet et al., 2017; Rivetti di Val Cervo et al., 2017; Singh et al., 2022; Livingston et al., 2024), but also exerts anti-inflammatory effects and reduces inflammatory responses in the injured area (Guo et al., 2014; Liu et al., 2020; Chen et al., 2023).

In this study, we overexpressed the transcription factor ND1 in astrocytes of the injured area using an adeno-associated virus in a mouse model of RIBI. Immunofluorescence staining and bulk RNA sequencing (RNA-seq) were used to assess the therapeutic effects and investigate the treatment mechanisms. Additionally, we conducted further validation in a mouse model of hemorrhagic stroke.

## Methods

### Animals

For the experiments, we only examined male C57BL/6J mice because males exhibit less variability in phenotype than females. The male C57BL/6J mice (8–10 weeks old, around 25 g) were purchased from Beijing Vital River Laboratory Animal Technology Co. Ltd. (Beijing, China, animal license No. SCXK 2022-0063) and were housed in a specific-pathogen-free environment with a maximum of five mice per cage, and at a temperature range of 21–26°C, relative humidity range of 40%–60%, and a light cycle of 8:00–20:00. All animals were allowed *ad libitum* access to food and water. The suffering of experimental animals was minimized. All animal experiments were performed in accordance with protocols approved by the Institutional Animal Care and Use Committee of Jinan University (Guangzhou, China, approval No. IACUC-20230602-12) on June 2, 2023.

### Construction of the radiation-induced brain injury mouse model

After anesthesia with 1% pentobarbital (0.2 mL/25 g), mice were fixed onto a stereotactic frame (Reward, Shenzhen, China). The GE Revolution computed tomography system (GE HealthCare, Chicago, IL, USA) was used to determine the brain regions that required radiation modeling (He et al., 2020) (computed tomography energy: 80 kV; imaging range: 250 mm; layer thickness: 0.625 mm; pixel size: 0.49 mm × 0.49 mm). Subsequently, the Leksell Gamma Knife Perfexion (Elekta, Stockholm, Sweden) was used to irradiate mice. The left thalamic region of the mouse was irradiated with gamma rays with a single dose of 50 Gy (anterior-posterior, –2 mm; lateral, 2 mm; dorsal-ventral, 2 mm). The brain parenchymal lesion caused by radiation was observed by MRI and immunofluorescence staining.

### Magnetic resonance imaging

MRI was performed on the mice at 2 and 10 weeks after irradiation. Mice were rapidly anesthetized with 3% isoflurane/oxygen (Reward), and then maintained under anesthesia at a concentration of 1%. The T2 signal of the lesion was imaged using a 7T MRI device (Bruker Biospec, Karlsruhe, Germany) with Paravision 6.0 operating system, and the lesion volume of mice was measured by ITK-SNAP software (version 3.8.0, University of North Carolina, Raleigh, NC, USA).

### Construction of the hemorrhagic stroke model

The mouse model was established as previously described (Zeng et al., 2017; Yu et al., 2020; Shi et al., 2023b). Mice were anesthetized with 1.25% tribromoethanol (0.2 mL/10 g; Sigma Aldrich, St. Louis, MO, USA) intraperitoneally (Hill et al., 2013). Type IV collagenase (0.5 μL, 0.06875 U/μL, 17104019, Gibco, Grand Island, NY, USA) was injected *in situ* into the thalamus (anterior–posterior, –1.46 mm; medial–lateral, 1.25 mm; dorsal–ventral, –3.5 mm). Because a cavity formed in the core area of the thalamic injury, to avoid statistical errors, we conducted high-magnification photography using a laser scanning confocal microscope (LSM780, Zeiss, Oberkochen, Germany) and statistical analysis within a 1-mm^2^ area adjacent to the cavity.

### Stereotaxic virus injection

Two weeks after the establishment of the RIBI model (1 week after the establishment of the hemorrhagic stroke model), mice were randomly divided into two groups: control virus and ND1-treated groups, with 12 mice in each group. All mice were anesthetized with intraperitoneal injection of 1.25% tribromoethanol (0.2 mL/10 g). Virus (2 μL) was injected into the thalamus. The ND1-treated mice were injected with AAV9 GFAP104::mND1-tdTomato (5320, PackGene Biotech, Guangzhou, China) at a virus concentration of 5.0E+11 GC/mL, and the control virus mice were injected with AAV9 GFAP104::tdTomato (10061, PackGene Biotech) at a virus concentration of 5.0E+11 GC/mL. AAV9 virus was used because of its ability to cross the BBB. Furthermore, this virus infects neurons and astrocytes in the central nervous system, with its tissue specificity determined by the promoter. Adding the GFAP104 promoter (which consists of an EF1α enhancer followed by the gfaABC1D promoter) (Xu et al., 2023), at a virus titer of 5.0E+11 GC/mL (Xu et al., 2022), can achieve more specific expression in astrocytes, thereby enhancing the specificity of transcription factor expression.

### Immunofluorescence staining

The RIBI mice were sampled and subjected to immunofluorescence staining at 11 weeks after modeling. Before sampling, the mice were anesthetized by intraperitoneal injection of 1.25% tribromoethanol (0.2 mL/10 g), and then perfused with 0.9% saline and 4% paraformaldehyde solution. The brains were fixed with paraformaldehyde, dehydrated with 20% and 30% sucrose, and then frozen. The tissue was then sectioned with a freezing microtome (CRYOSTAR NX50, Thermo Scientific, Waltham, MA, USA) at –20°C at a thickness of 30 μm. The prepared brain slices were first blocked with 5% blocking serum (donkey species, SL050, Solarbio, Beijing, China), then incubated with primary antibodies overnight (4°C). The next day, the brain slices were washed with 0.01 M phosphate buffer saline, followed by incubation with the appropriate fluorescent secondary antibodies at room temperature for 2 hours, and then 4′,6-diamidino-2-phenylindole (70508621, Roche, Basel, Switzerland). After incubation, the brain slices were attached to an adhesive glass slide and sealed with a polyvinyl alcohol sealing agent (10981, Merck, Darmstadt, Germany). The antibody information is shown in **Table 1**.

**Table 1:** Antibodies for immunostaining

Antibody	Species	Dilution	Company	Cat#	RRID
GFAP	Rat	1:1000	Invitrogen, Carlsbad, CA, USA	13-0333	AB_86543
S100β	Rabbit	1:1000	Abcam, Cambridge, UK	AB52642	AB_882426
ND1	Rabbit	1:1000	Abcam	AB205300	AB_3083561
NeuN	Guinea pig	1:1000	Millipore, Bedford, MA, USA	ABN90	AB_11205592
RFP	Rat	1:1000	Chromo Tek, Munich, Germany	5F8	AB_2336064
Iba1	Rabbit	1:1000	Wako, Tokyo, Japan	019-19741	AB_839504
CD68	Rat	1:1000	Invitrogen	14-0681-82	AB_2572857
AQP4	Rabbit	1:1000	Proteintech, Chicago, IL, USA	16473-1-AP	AB_2827426
CD31	Goat	1:1000	Bio-Techne, Minneapolis, MN, USA	AF3628	AB_2161028
CD8	Rabbit	1:1000	Abcam	AB217344	AB_2890649
Anti-rabbit Alexa Fluor 488	Donkey	1:1000	Thermo Scientific, Waltham, MA, USA	A21206	AB_2535792
Anti-goat Alexa Fluor 488	Donkey	1:1000	Thermo Scientific	A11055	AB_2534102
Anti-rat Alexa Fluor 555	Donkey	1:1000	Jackson Immunoresearch Laboratories, West Grove, PA USA	712-165-150	AB_2340666
Anti-guinea pig Alexa Fluor 647	Donkey	1:1000	Jackson Immunoresearch Laboratories	706-605-148	AB_2340476
Anti-rabbit Alexa Fluor 647	Donkey	1:1000	Thermo Scientific	A31573	AB_2536183
Anti-rat Alexa Fluor 647	Goat	1:1000	Thermo Scientific	A21247	AB_141778

AQP4: Aquaporin-4; CD68: cluster of differentiation 68; GFAP: glial fibrillary acidic protein; Iba1: ionized calcium binding adapter molecule 1; ND1: NeuroD1; NeuN: neuronal nuclei; RFP: red fluorescent protein; S100β: S100 calcium-binding protein beta.

After immunofluorescence staining, brain slices were imaged at 10×, 20×, 40× and 63× by an automated upright fluorescence microscope (Axio Imager Z2, Zeiss) and a laser scanning confocal microscope (LSM780, Zeiss). When imaging at 20×, 40× and 63×, the Z-axis thickness was 16 μm and the interlayer spacing was 1 μm. At least three mice per group were used for data collection and statistical analysis in every experiment. We analyzed the neuronal density and colocalization in the thalamus images at 20× magnification with 16-μm-thick stacks. Most immunofluorescence images were processed by the Zeiss software ZEN (version: 3.3.89, Zeiss), and data acquisition and analysis were performed using ImageJ software (version: 1.54f, National Institutes of Health, Bethesda, MD, USA) (Schneider et al., 2012).

For cell counting, mouse brains were processed and stained in frozen sections (30 μm thickness), and the thalamus was photographed using a laser confocal microscope at 20× magnification, with an area of 1.28 mm^2^, a layer scan thickness of 16 μm, and an interlayer spacing of 1 μm. The colocalization of Iba1^+^ and DAPI^+^ cells was recorded in 40× images. Positive cells were counted layer by layer using ImageJ software.

### Sholl analysis

To assess the morphological characteristics of microglia, we used the Sholl Analysis Plugin, which was developed by the Ghosh lab (Cambridge, MA, USA). In brief, we superimposed a series of concentric rings, with a diameter difference of either 5 or 10 μm, emanating from the center of the microglia soma onto each microscopic image. Subsequently, we counted the number of branches that crossed each of these concentric rings.

### RNA sequencing

The thalamus was isolated and subjected to RNA-seq. After perfusion with saline, the brains of the mice were removed on ice and sectioned using a mouse coronal cutting mold with 1-mm intervals to obtain the section from approximately anterior–posterior –1.23 mm to –2.45 mm. The thalamus tissue located beneath the hippocampus was carefully removed using tweezers and placed into an RNase-free EP tube (AM12400, Thermo Scientific). After rapid freezing in liquid nitrogen, the samples were stored in a –80°C freezer and sent to a sequencing company for analysis.

The brain tissue RNA was extracted using the Trizol Reagent (15596018, Invitrogen, Carlsbad, CA, USA) as per the manufacturer’s protocol. Then, the mRNA was reverse transcribed into complementary DNA using NEBNext Ultra RNA Library Prep Kit for Illumina (NEB 7530, New England Biolabs, Ipswich, MA, USA). The ligation reaction was purified with the AMPure XP Beads (1.0×) and subjected to amplification by polymerase chain reaction. The prepared complementary DNA library was then sequenced using Illumina Novaseq6000 by Gene Denovo Biotechnology Co. (Guangzhou, China). Bioinformatics analysis was conducted on the sequencing data to ensure the generation of high-quality data for downstream analysis.

Differential gene expression analysis was carried out using DESeq2 (Bioconductor, Cambridge, MA, USA). Genes with a false discovery rate below 0.05 and an absolute fold change ≥ 2 were considered differentially expressed. Gene Set Enrichment Analysis (GSEA) was performed using the Molecular Signatures Database to evaluate significant differences in specific Gene Ontology terms, Kyoto Encyclopedia of Genes and Genomes pathways, and Reactome pathways between the two groups. Soft clustering analysis was applied to identify expression patterns in three groups, determining the optimal cluster number and setting a membership threshold of 0.6. Functional annotation analysis of each cluster was performed using the online tool g: Profiler1 (https://www.omicshare.com). Protein–protein interaction networks were identified using String v10 (Gene Denovo, Guangzhou, China), visualized in Cytoscape (version: v3.7.1, Gene Denovo) to highlight core and hub gene biological interactions.

Next-generation sequencing data generated in this study were deposited in the Gene Expression Omnibus database of National Center for Biotechnology Information (Gene Expression Omnibus accession: GSE273938) for RNA-Seq.

### Statistical analysis

All data were analyzed by GraphPad Prism 7.00 (GraphPad Software, Boston, MA, USA, www.graphpad.com) and are presented as mean ± standard deviation except the data from the Sholl analysis, which are presented as mean ± standard error of the mean. Comparisons between two groups were performed by unpaired Student’s *t*-test. For comparisons between multiple groups with a single factor, one-way analysis of variance was performed. For pairwise comparisons, *post hoc* tests were performed with Tukey’s correction. Data from the Sholl analysis were analyzed by two-way repeated measure analysis of variance. *P* < 0.05 was considered statistically significant.

## Results

### Reduction of radiation-induced brain lesion volume and increase of neuronal density after NeuroD1 treatment

**Figure 1:**
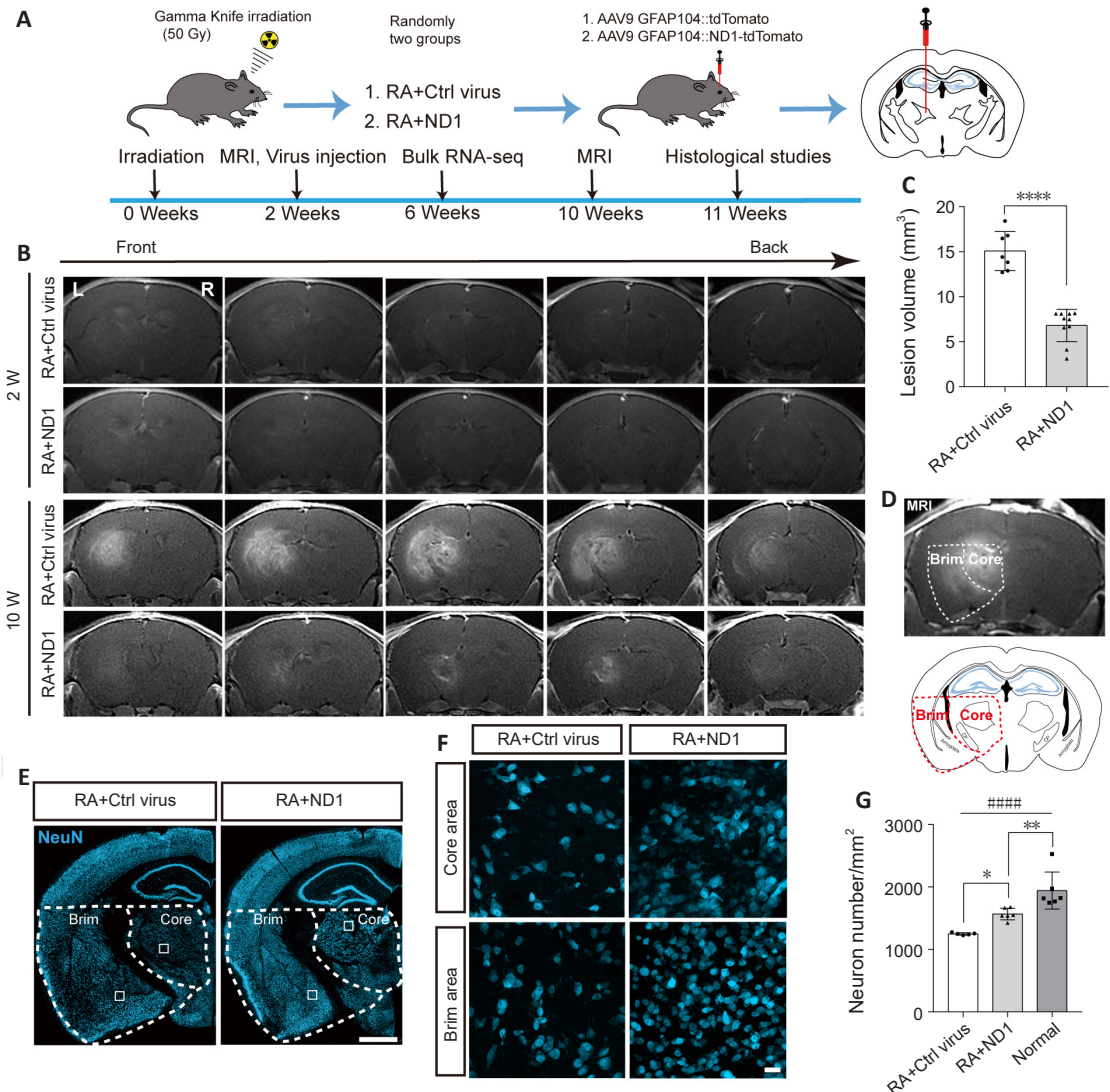
Reduction of radiation-induced brain lesion volume and increase of neuronal density after ND1 treatment. (A) Schematic diagram illustrating experimental timeline. (B) Representative MRI images illustrating radiation injury (white signal) at 10 weeks after radiation (bottom two rows) but not at 2 weeks after radiation (top two rows). The ND1 group had a smaller lesion volume than that in the control group at 10 weeks after radiation. (C) Quantitative analysis of lesion volume (*n* = 7–10). (D) Representative MRI images of core and brim areas after radiation. (E) Representative images of NeuN (blue, neuronal marker, Alexa Fluor ® 647) signal in core or brim areas. ND1-treated mice had more neurons than control virus mice. Scale bar: 1000 μm. (F) Confocal images (40× magnification) showing a representative enlarged view of the NeuN density under different conditions. ND1-treated mice had increased NeuN density compared with that in control virus mice. Scale bar: 20 μm. (G) Quantitative analysis of NeuN density after ND1 treatment (*n* = 5–6). Data are expressed as mean ± SD. *P < 0.05, **P < 0.01, ****P < 0.0001 (unpaired Student’s *t*-test [C], ordinary one-way analysis of variance followed by Tukey’s *post hoc* test [G]). MRI: magnetic resonance imaging; ND1: NeuroD1; RA: radiation; RA + ND1: radiation + NeuroD1; RA + Ctrl virus: radiation + control virus; RNA-seq: RNA sequencing.

To investigate the therapeutic effect of *in situ* neuronal regeneration in an RIBI mouse model, we used MRI at 2 weeks and 10 weeks after modeling. Bulk RNA-seq was performed at 6 weeks after modeling. Histology evaluation was performed on samples collected at 11 weeks post-modeling (**Figure 1A**). The MRI results showed no significant radiation injury lesions in either group at 2 weeks after modeling. However, the lesion volume in the ND1-treated group was significantly smaller than that in the control virus group at 10 weeks after modeling (**Figure 1B** and **C**).

We performed immunostaining for GFAP, a marker of reactive astrocytes (Galland et al., 2019), which showed that the GFAP-positive area was significantly larger in the control virus group than in the ND1-treated group at 11 weeks after modeling (**Additional Figure 1A** and **B**). On the basis of the MRI and GFAP staining results, we divided the radiation injury region into a core area and a brim area. The core area was defined as the thalamic region below the hippocampus, and the brim area included the cortical area lateral to the core area and below the lower edge of the hippocampus (**Figure 1D** and **Additional Figure 1C**). GFAP^+^ reactive astrocytes were spreading in both the core and brim areas in the control virus group, whereas in the ND1 group, GFAP^+^ reactive astrocytes were observed in only the core area (**Additional Figure 1A**).

Subsequently, we measured the density of NeuN (a marker for neurons) in the core and brim areas of the thalamus in both groups (**Figure 1E** and **F**). The neuronal density in the control virus group, ND1-treated group and normal mice were significantly different (**Figure 1G**); the neuronal density in the control virus group was significantly lower than that in the ND1-treated group. However, the neuronal density in the ND1-treated group was still significantly lower than that in normal mice (**Figure 1G**). These results suggest that ND1 treatment reduced the lesion volume and increased the neuronal density in the lesioned area of the RIBI mouse model.

### NeuroD1 mediates astrocyte-to-neuron conversion

**Figure 2:**
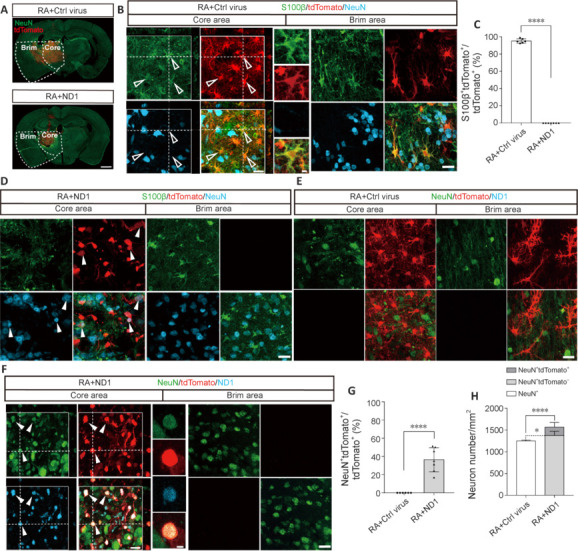
ND1 mediates astrocyte-to-neuron conversion. (A) Representative images of NeuN (green, Alexa Fluor ® 647) and virus (red, tdTomato+, Alexa Fluor ® 555) signals in core or brim areas. The tdTomato expression range in the control virus group was different than that in the ND1-treated group. Scale bar: 1000 μm. (B) Confocal images (63× magnification) showing enlarged views of the S100β (green, astrocytic marker, Alexa Fluor ® 488), tdTomato (red, tag protein of virus, Alexa Fluor ® 555) and NeuN (blue, Alexa Fluor ® 647) in different areas of control virus group. Typical images showing core area of control group (GFAP::tdTomato), tdTomato^+^ cells (red) co-stained with S100β^+^ astrocytes (green). Hollow arrows indicate S100β and tdTomato co-staining. Scale bars: 20 μm, 5 μm (enlarged images). (C) Quantitative analysis of percentage of S100β^+^tdTomato^+^ astrocytes (*n* = 6–7). (D) Confocal images (63× magnification) showing enlarged views of S100β (green, Alexa Fluor ® 488), tdTomato (red, Alexa Fluor ® 555) and NeuN (blue, Alexa Fluor ® 647) in different areas of the ND1-treated group. In the ND1-treated mice, there was no tdTomato expression in the lesion brim area. Solid arrows indicate NeuN and tdTomato co-staining. Scale bar: 20 μm. (E) Confocal images (63× magnification) showing enlarged images of NeuN (green, Alexa Fluor ® 647), tdTomato (red, Alexa Fluor ® 555) and NeuroD1 (blue, Alexa Fluor ® 488) in different areas of the control group. The brim area had less activated astrocytes than the core area. Scale bar: 20 μm. (F) Typical images showing tdTomato^+^ cells (red, Alexa Fluor ® 555) co-stained with NeuN^+^ neurons (green, Alexa Fluor ® 647) and NeuroD1 (blue, Alexa Fluor ® 488) in the core area of the ND1-treated group. In ND1-treated mice, there was no expression of NeuroD1 or tdTomato in the lesion brim area. Solid arrows indicating NeuN, tdTomato and NeuroD1 co-staining. Scale bars: 20 μm, 5 μm (enlarged images). (G) Quantitative analysis of percentage of NeuN^+^tdTomato^+^ neurons (*n* = 6–7). (H) Quantification of total NeuN^+^ cells under different conditions (*n* = 5–6). There were more non-converted neurons in the ND1-treated group (NeuN^+^tdTomato^−^) than in the control group. Data are expressed as mean ± SD. *P < 0.05, ****P < 0.0001 (unpaired Student’s *t*-test). ND1: NeuroD1; RA: radiation; RA + ND1: radiation + NeuroD1; RA + Ctrl virus: radiation + control virus.

To confirm whether *in situ* neuronal regeneration converts reactive astrocytes into neurons by overexpressing ND1 transcription factor in astrocytes of the RIBI mouse model, we examined the expression of tdTomato (a fluorescent protein for adeno-associated virus), S100 calcium-binding protein B (S100β, a marker for astrocytes) (Galland et al., 2019), NeuN (Gusel’nikova and Korzhevskiy, 2015) and ND1. In the control virus group, tdTomato expression was spreading in both the core and brim areas (**Figure 2A**, top panel), while, in the ND1-treated group, tdTomato expression was observed in only the core area (**Figure 2A**, bottom panel), similar to the pattern found with GFAP^+^ reactive astrocytes shown in **Additional Figure 1A**. In addition, in the control virus group, the tdTomato^+^ cells in both the core and brim areas (**Figure 2B**) colocalized with S100β at a rate of 95.2% ± 2.5% (**Figure 2C**), and did not colocalize with NeuN. The left two columns of **Figure 2B** show a confocal orthogonal view, providing more details of the colocalization of S100β and tdTomato. By contrast, in the ND1-treated group, tdTomato^+^ cells in the core area did not colocalize with S100β (**Figure 2D**, left two columns) and there was no tdTomato expression in the brim area (**Figure 2D**, right two columns). These results indicate that tdTomato^+^ cells in the lesion areas of the control virus group were still astrocytes, whereas tdTomato^+^ cells in the ND1-treated areas were no longer astrocytes at 11 weeks after RIBI modeling.

No ND1 protein expression was observed in either the core or brim areas (**Figure 2E**) in the control virus group. In the ND1-treated mice, there was no expression of ND1 or tdTomato in the brim area of the lesion (**Figure 2F**, right two columns). However, tdTomato^+^ cells in the core area colocalized with both ND1 and NeuN (**Figure 2F**, left two columns), with a colocalization (NeuN^+^tdTomato^+^) rate of 36.4% ± 13.2% (**Figure 2G**). In the control virus mice, tdTomato^+^ cells did not colocalize with NeuN (**Figure 2G**). These results suggest that ND1 overexpression converted tdTomato^+^ astrocytes into NeuN^+^ neurons.

We further analyzed the number of transdifferentiated neurons and endogenous neurons in the different groups. As shown in **Figure 2H**, the endogenous neuronal density in the lesion area of the ND1-treated mice (NeuN^+^tdTomato^–^) was significantly higher than that of the control virus group (NeuN^+^). The total neuronal density in the ND1-treated mice increased to 1568 ± 93.1 cells/mm^2^, which was significantly higher than that of the control virus group mice, but lower than the 1944 ± 297.2 cells/mm^2^ in normal mice (**Figure 1G**). These results suggest ND1 overexpression not only induced transdifferentiation but also protected the endogenous neurons.

**Figure 3:**
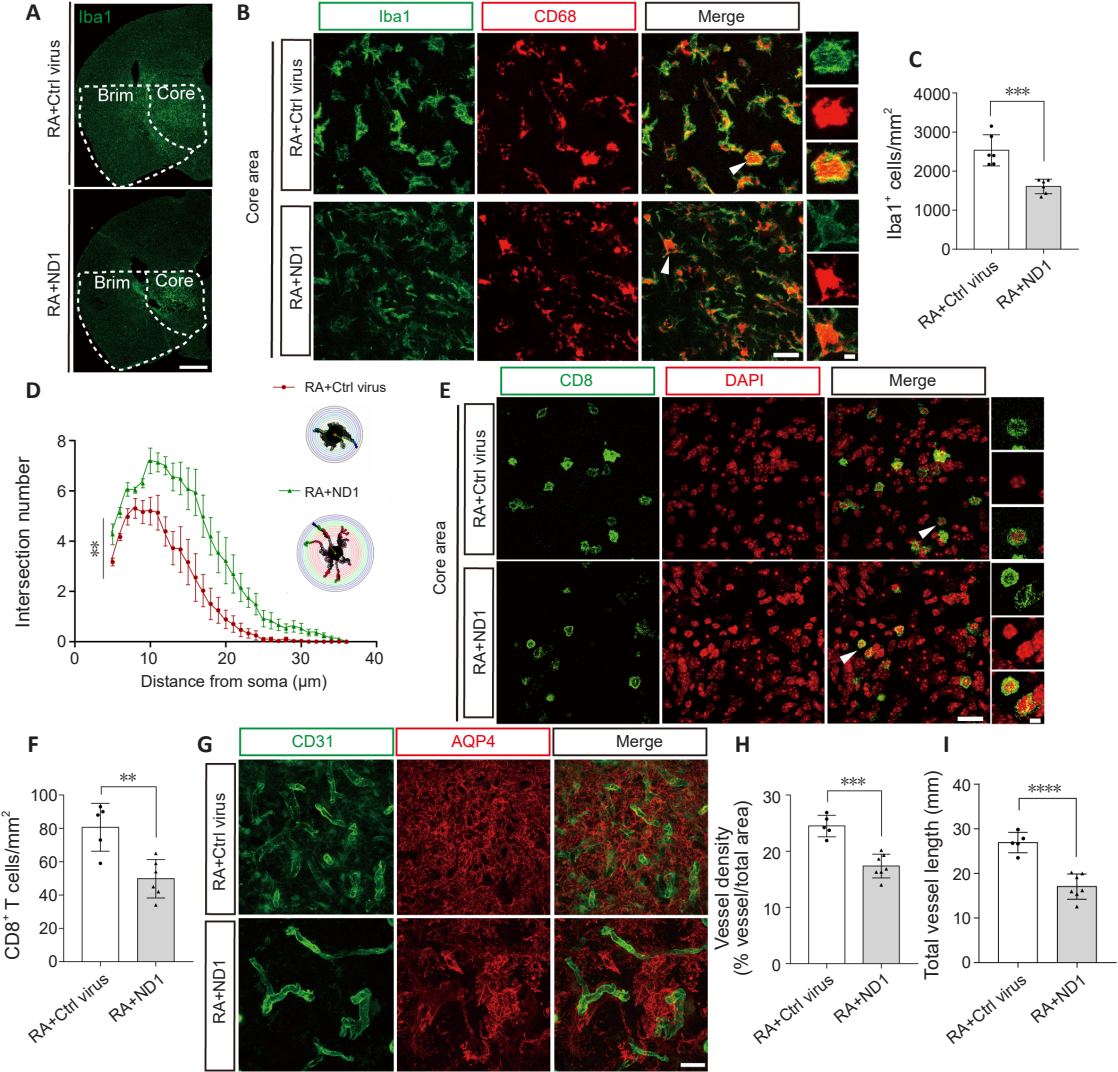
ND1 overexpression improves the microenvironment of the injured core area in the RIBI mouse model. (A) Representative images of microglia-occupied areas (Iba1^+^, green, Alexa Fluor® 488). The control virus group had a larger microglia-occupied area than the ND1-treated group. Scale bar: 1000 μm. (B) Confocal images (63× magnification) showing enlarged views of Iba1 (green, microglial marker, Alexa Fluor® 488) and CD68 (red, activated microglial marker, Alexa Fluor® 647) under different conditions. The density of Iba1^+^ cells were different between the two groups. The white arrows indicate typical activated microglia cells. Scale bars: 20 μm, 5 μm (enlarged images). (C) Quantitative analysis of Iba1 density in core areas (*n* = 6). (D) Sholl analysis result showed microglia in ND1-treated group had more branches than the control virus group, suggesting less reactive (*n* = 6). (E) Confocal images (63× magnification) of CD8^+^ T cells (green, Alexa Fluor® 647). The control virus group had more CD8^+^ T cells than the ND1-treated group. White arrows indicate typical CD8^+^ T cells. Scale bars: 20 μm, 5 μm (enlarged images). (F) Quantitative analysis of the number of CD8^+^ T cells per 1 mm^2^ in core areas (*n* = 5–6). (G) Confocal images (40× original magnification) of CD31 (green, vascular endothelial cell marker, Alexa Fluor ® 488 and AQP4 (red, astrocytic endfoot marker, Alexa Fluor® 647) under different conditions. The control virus group had more vessels than the ND1-treated group. Scale bar: 20 μm. (H) Quantitative analysis of vessel density in the core area (*n* = 5–7). (I) Quantitative analysis of total vessel length in the core areas (*n* = 5–7). Data are expressed as mean ± SD. **P < 0.01, ***P < 0.001, ****P < 0.0001 (unpaired Student’s *t*-test [C, F, H, I], two-way repeated measure analysis of variance [D]). ND1: NeuroD1; RA: radiation; RA + ND1: radiation + NeuroD1; RA + Ctrl virus: radiation + control virus.

To clarify the neuroprotective effect of ND1 overexpression, we further investigated the microenvironment around the injured neurons in the RIBI mice. First, we observed the effect of ND1 overexpression on microglial activation. **Figure 3A** shows the expression of ionized calcium binding adaptor molecule 1 (Iba1), a marker for microglia (Hendrickx et al., 2017), in the core and brim areas of the lesion in different groups. As shown in **Figure 3B** and **D**, the morphology of the microglia in the control virus group were often round in shape (top row), suggesting reactive, whereas in the ND1-treated group, microglia had more branches (bottom row), suggesting less reactive (*P* = 0.0099, two-way repeated measure analysis of variance). The density of Iba1^+^ cells in the core area of the ND1-treated group was significantly higher than that in the control virus group (**Figure 3C**). Moreover, the mean fluorescence intensities of both Iba1 and cluster of differentiation 68 (CD68), a marker for microglia activation (Hendrickx et al., 2017), in the core area and the brim area of the control virus group were significantly higher than those in the ND1-treated group (**Additional Figure 1D–H**). These results suggest that ND1 overexpression decreased microglial activation in the lesion regions of RIBI mice.

Given that our previous study showed an increase in cytotoxic CD8^+^ T cell infiltration mediated by microglial activation in the lesion area of mice with RIBI (Shi et al., 2023a), we subsequently examined the infiltration of CD8^+^ T cells in the brain parenchyma (**Figure 3E**). The density of CD8^+^ T cells in the thalamus of the control virus group was 80.6 ± 14.3 cells/mm^2^, significantly higher than 49.8 ± 11.6 cells/mm^2^ in the ND1-treated mice (**Figure 3F**). These results suggest that ND1 overexpression decreased the infiltration of CD8^+^ T cells in the lesion regions of RIBI mice.

In addition to microglial activation and CD8^+^ T cell infiltration, BBB disruption is a typical pathological feature of RIBI (Ali et al., 2019). Radiation increases the release of vascular endothelial growth factor and causes incomplete angiogenesis, which disrupts the BBB (Cheng et al., 2023). Therefore, we examined blood vessels using aquaporin 4 (AQP4), which is located in the astrocyte endfoot and wraps around blood vessels, contributing to the BBB (Nagelhus and Ottersen, 2013), and platelet endothelial cell adhesion molecule-1 (PECAM-1/CD31), a marker for vascular endothelial cells. **Figure 3G** shows the co-staining of AQP4 and CD31 in the core area in different groups. We observed microvascular dilation in the injury site of the RIBI mice, consistent with our previous findings (He et al., 2020). The control virus group and ND1-treated group exhibited a loss of AQP4 polarity, with AQP4 no longer wrapping around the blood vessels. Moreover, the vascular density and total vascular length were significantly higher in the control virus group than in the ND1-treated group (**Figure 3H** and **I**). There were no significant differences in vascular density and total vascular length between the two groups in the brim area (**Additional Figure 1I–K**). These results suggest that ND1 treatment reduced angiogenesis in the RIBI model mice, thereby improving the BBB integrity.

### RNA sequencing in the irradiated brain tissue after NeuroD1 virus injection

**Figure 4:**
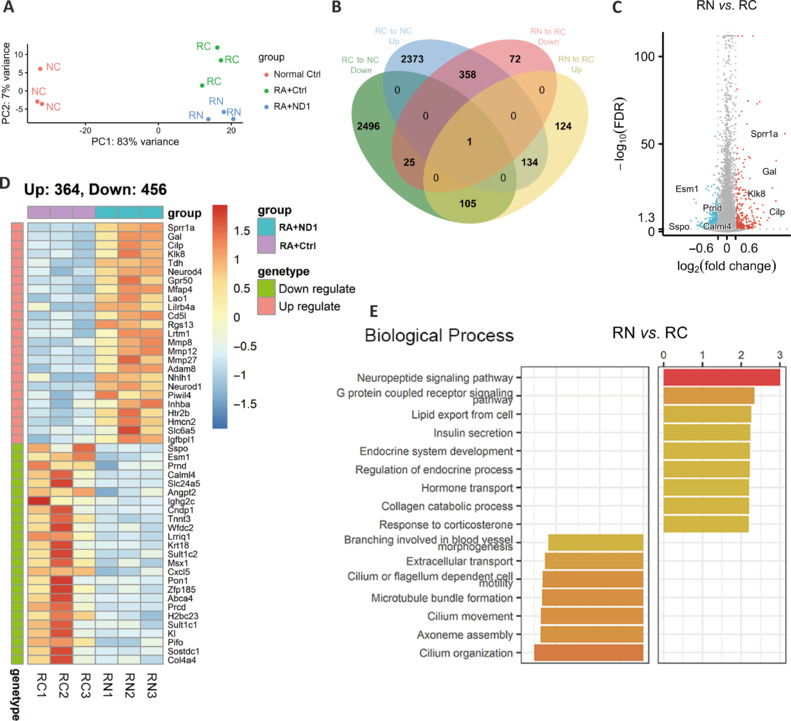
RNA sequencing in the irradiated brain tissue after NeuroD1 virus injection. (A) Principal component analysis showed the relationship of the different samples. (B) The Venn diagram indicates the overlap of differentially expressed genes between different groups. (C) The volcano diagram shows upregulated genes (red) and downregulated genes (blue) in the RN *vs.* RC groups. (D) A heat map demonstrating significant differential gene expression between the RN and RC groups. (E) Enrichment of related biological process by Gene Set Enrichment Analysis between the RN and RC groups (*n* = 3). NC: Normal control mice; RC: radiation + control virus; RN: radiation + NeuroD1 virus.

To elucidate the molecular mechanisms underlying the therapeutic effects achieved by ND1 overexpression, we conducted bulk RNA-seq on brain tissue at 6 weeks after RIBI mice modeling. Principal component analysis showed that normal control mice (NC) were relatively distant from both RIBI mice treated with control virus (RC) and ND1 virus (RN), indicating severe injury of the model mice after radiation (**Figure 4A**). Within each group (NC, RC, and RN), the mice were closely clustered, indicating consistent gene expression patterns within each individual group. The genes with a false discovery rate below 0.05 and an absolute fold change of ≥ 2 were considered differentially expressed. A total of 364 genes were significantly upregulated and 456 genes were downregulated in the RN group compared to the RC group. Compared to the NC group, 2866 genes were significantly upregulated and 2627 genes were downregulated in the RC group (**Figure 4B**). The volcano plot provided an overview of significantly upregulated (red) and downregulated (blue) genes in the RN versus RC group (**Figure 4C**).

A heatmap clustering of the top 25 most significantly upregulated and downregulated genes was shown in **Figure 4D**. Of the top 25 significantly upregulated genes in the comparison between RN and RC, *NeuroD4* and *NeuroD1*, *Sprr1a*, *Ga*l, *Klk8*, *Rgs13*, *Lrtm1*, *Adam8*, *Nhlh1*, and *Piwil4* are all associated with neurogenesis. Of the top 25 significantly downregulated genes, *Ighg2* and *Cxcl5* are related to immune response; *Esm1* and *Angpt2* are related to angiogenesis; and *Pon1* and *Prcd* are related to apoptosis. The sequencing results aligned with our immunofluorescence staining findings, where increased neuronal density, decreased incomplete angiogenesis and reduced inflammatory response were observed in the ND1-treated mice. To investigate the cellular processes that were altered with ND1 viral treatment in RIBI mice, we performed GSEA on differential genes between groups. Of the biological process-related terms, significantly upregulated pathways included the neuropeptide signaling pathway, and significantly downregulated pathways included blood vessel branching morphogenesis (**Figure 4E**). These results suggest that neurogenesis, immune response and angiogenesis are potential biological process targets of ND1 overexpression for further study in RIBI.

### The dynamic changes and interaction of differentially expressed genes after NeuroD1 overexpression

**Figure 5:**
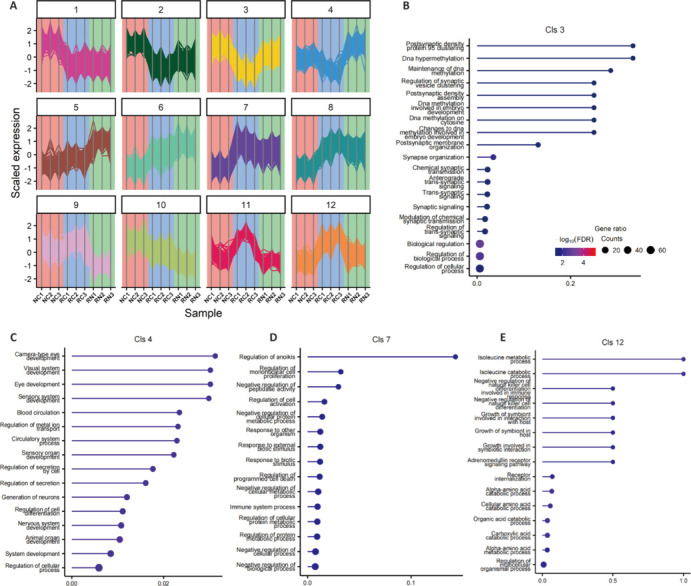
Dynamic changes and interaction of differentially expressed genes after ND1 overexpression. (A) Twelve distinct clusters with different change trends were screened out. (B–E) Functional annotations were conducted for clusters 3, 4, 7 and 12 (*n* = 3). ND1: NeuroD1.

The expression trends of genes with similar expression patterns in each group were analyzed using soft clustering analysis (**Figure 5A**), and 12 clusters with distinct expression patterns were screened out. Functional annotations for these clusters are presented in **Figure 5B–E**. Genes in clusters 3 and 4 (**Figure 5B** and **C**) demonstrated a gradual decreasing trend in the RC group and a gradual increasing trend in the RN group, related to cellular development and synaptic function. Clusters 7 and 12 (**Figure 5D** and **E**) showed trends of an increase in gene expression in the RC group and a decrease in the RN group, associated with immune response and negative regulation of cellular processes.

A protein–protein interaction network for differentially expressed genes between the RN and RC groups was conducted to elucidate the biological interactions of core and key genes (**Additional Figure 2**). Genes directly connected to ND1 were primarily upregulated (red circles), indicating an upregulation of cell differentiation-related genes. Additionally, ND1 indirectly influenced the regulation of immune response-related genes and tubular structure development-related genes (green and blue circles). These results from bulk RNA-seq suggest that ND1 overexpression modulated the expression of genes involved in neurogenesis, immune response, and angiogenesis, corresponding to observed phenotypes of ND1 treatment in RIBI.

### *In vivo* conversion of astrocytes into neurons in the delayed brain injury of a hemorrhagic stroke mouse model

**Figure 6:**
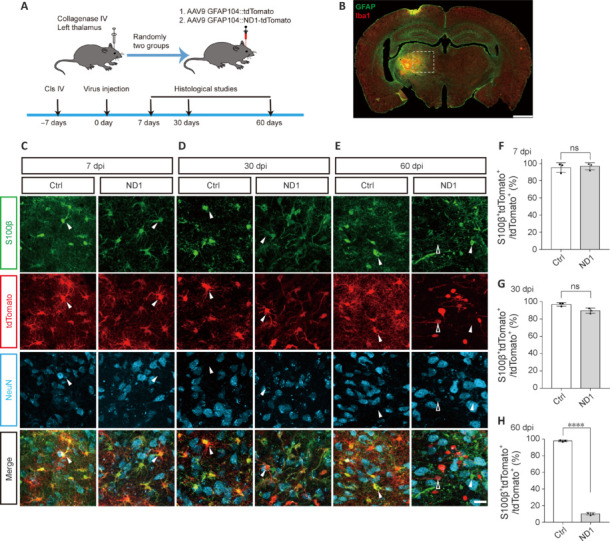
*In vivo* conversion of astrocytes into neurons in the thalamus of hemorrhagic stroke mouse brain. (A) Schematic diagram illustrating experimental timeline. (B) Representative images of reactive astrocyte (GFAP^+^, astrocytic marker, green, Alexa Fluor® 647) or microglia (Iba1^+^, microglial marker, red, Alexa Fluor® 488) -occupied areas on the 7^th^ day after injection with collagenase IV. Confocal images were taken from selected regions within a 1-mm^2^ area near the core injury area (dashed box). Scale bar: 1000 μm. (C–E) Confocal images (63× magnification) showing enlarged views of the S100β (green, astrocytic marker, Alexa Fluor® 488), tdTomato (red, tag protein of virus, Alexa Fluor ® 555) and NeuN (blue, neuronal marker, Alexa Fluor® 647) in the control group (left column) and ND1-treated group (right column) at 7, 30 and 60 days after viral injection. Astrocytes in the ND1-treated group gradually converted into neurons. Solid arrows indicate typical S100β^+^ cells. Hollow arrows indicate tdTomato and NeuN co-staining. Scale bar: 20 μm. (F–H) Quantitative analysis of percentage of tdTomato^+^ cells in both groups. Data are expressed as mean ± SD (*n* = 3). ****P < 0.0001 (unpaired Student’s *t*-test). Ctrl: Control; dpi: days post-virus injection; GFAP: glial fibrillary acidic protein; Iba1: ionized calcium binding adapter molecule 1; ND1: NeuroD1; ns: not significant.

Hemorrhagic stroke is an acute cerebrovascular disease in which brain tissue is damaged because of sudden obstruction of blood flow or rupture of blood vessels in the brain tissue, which subsequently leads to delayed brain injury (Ohashi et al., 2023). Histopathology evaluations were performed at 7, 30, and 60 days post-virus injection (dpi) (**Figure 6A**) within a 1-mm^2^ area adjacent to the cavity (**Figure 6B**). We performed co-staining for S100β, tdTomato and NeuN to examine the conversion of the astrocytes into neurons after overexpressing ND1 in the control virus group (Ctrl) and ND1-treated group (ND1).

At 7 dpi, the tdTomato^+^ cells were co-labeled with S100β but not with NeuN in both the Ctrl and ND1 groups (**Figure 6C** and **F**), which indicated that the tdTomato^+^ cells were still astrocytes at 7 dpi. At 30 dpi, tdTomato^+^ cells in the Ctrl group were still co-labeled with S100β but not with NeuN (**Figure 6D** and **G**). In the ND1 group, although most of the tdTomato^+^ cells were still co-labeled with S100β and not with NeuN, the co-labeling rate (89.7% ± 3.0%) was slightly lower compared with that at 7 dpi (96.8% ± 4.1%). These results suggest a trend that a small proportion of tdTomato+ cells had become non-astrocytes at 30 dpi in the ND1-treated group.

At 60 dpi, tdTomato^+^ cells were co-labeled with S100β in the Ctrl group. In the ND1 group, tdTomato^+^ cells were sparsely co-labeled with S100β and were co-labeled with NeuN. These results suggest most of the tdTomato^+^ cells in the ND1-treated group had become non-astrocytes, with some of them converting into neurons at 60 dpi (**Figure 6E** and **H**). These results suggest that the tdTomato^+^ astrocytes gradually converted into neurons after ND1 overexpression.

**Figure 7:**
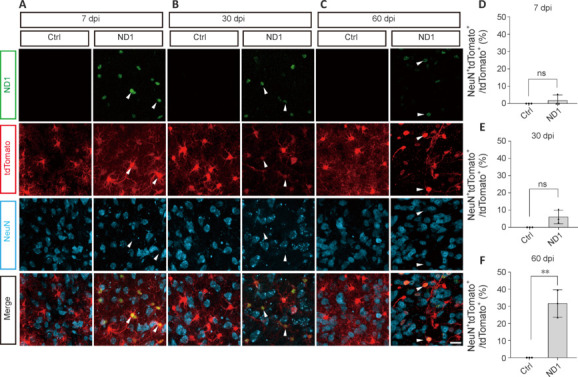
Reactive astrocytes gradually converted into neurons by NeuroD1. (A–C) Confocal images (63× magnification) of the NeuroD1 (green, Alexa Fluor® 488), tdTomato (red, tag protein of virus, Alexa Fluor® 555) and NeuN (blue, neuronal marker, Alexa Fluor® 647) in the control and ND1-treated groups at 7, 30 and 60 dpi. In the ND1-treated group tdTomato^+^ cells gradually converted into neurons. Solid arrows indicate typical NeuroD1^+^ cells. Scale bar: 20 μm. (D–F) Quantitative analysis showing no tdTomato and NeuN co-stained cells in the control group and gradually increasing co-stained cells in the ND1-treated group at 7 (1.8% ± 3.1%), 30 (6.0% ± 3.9%) and 60 (31.7% ± 8.0%) dpi. Data are expressed as mean ± SD (*n* = 3). **P < 0.01 (unpaired Student’s *t*-test). Ctrl: Control; dpi: days post-virus injection; ND1: NeuroD1; ns: not significant.

To determine whether the conversion of tdTomato^+^ astrocytes into neurons was mediated by the increased expression of ND1, we performed co-staining for ND1, tdTomato and NeuN at 7, 30, and 60 dpi in a hemorrhagic stroke mouse model. In the Ctrl group, there was no ND1 expression and the tdTomato^+^ cells were not co-labeled with NeuN (**Figure 7A–F**). In the ND1 group, ND1 was expressed in tdTomato^+^ astrocytes and most of ND1 did not co-label with NeuN at 7 dpi (**Figure 7A**). The co-labeling rate of NeuN^+^tdTomato^+^ was 1.8% ± 3.1% (**Figure 7D**). At 30 dpi, there was a slight increase in the co-labeling rate of NeuN^+^tdTomato^+^ to 6.0% ± 3.9% (**Figure 7B** and **E**). At 60 dpi, tdTomato+ cells not only expressed ND1 but also were co-labeled with NeuN (**Figure 7C**), suggesting that these cells had transdifferentiated into neurons under the induction of ND1. The co-labeling rate of tdTomato^+^NeuN^+^ was 31.7% ± 8.0% (**Figure 7F**), which indicated that the transdifferentiation induced by ND1 was a gradual increase.

### Reduction of microglial activation after NeuroD1 treatment in a hemorrhagic stroke mouse model

**Figure 8:**
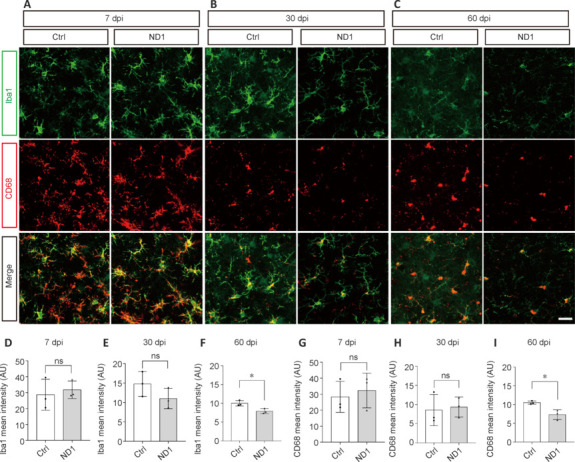
Reduction of inflammation after ND1 treatment in hemorrhagic stroke mouse brain. (A–C) Confocal images (63× magnification) of the Iba1 (green, microglial marker, Alexa Fluor® 488) and CD68 (tdTomato, activated microglial marker, Alexa Fluor ® 647) in the control and ND1-treated groups at 7, 30 and 60 dpi. Both groups had decreased inflammation from 7 to 60 dpi. Scale bar: 20 μm. (D, E) Quantitative analysis showing no significant difference of Iba1 mean intensity between the two groups at 7 and 30 dpi. (F) Quantitative analysis showing a significant reduction of Iba1 mean intensity after ND1 treatment compared with that in the control group at 60 dpi. (G, H) Quantitative analysis showing no significant difference of CD68 mean intensity between the two groups at 7 and 30 dpi. (I) Quantitative analysis showing a significant reduction of CD68 mean intensity after ND1 treatment compared with that in the control group at 60 dpi. Data are expressed as mean ± SD (*n* = 3). *P < 0.05 (unpaired Student’s *t*-test). Ctrl: Control; dpi: days post-virus injection; Iba1: ionized calcium binding adapter molecule 1; ND1: NeuroD1; ns: not significant.

The hemorrhagic stroke brain injury includes two characterized timespans: the early brain injury period and the delayed brain injury period. The microglial phenotypes and functions during each phase serve a critical role in both promoting and attenuating hemorrhagic stroke-induced morbidity (He et al., 2021; Alsbrook et al., 2023). We examined the activation level of microglial cells by co-staining the microglial markers Iba1 and CD68. The staining results showed that both groups of mice exhibited gradually reduced microglial activation over time (7, 30, and 60 dpi; **Figure 8A–C**). Compared with those in the Ctrl group, the mean fluorescence intensities of both Iba1 and CD68 gradually decreased after ND1 treatment (Iba1, **Figure 8D–F**; CD68, **Figure 8G–I**). Moreover, statistical analysis at 60 dpi showed that the mean fluorescence intensities of both Iba1 and CD68 in the ND1 group were significantly lower than those in the Ctrl group (**Figure 8C**, **F** and **I**). These results suggest that microglial activation in the ND1-treated mice was significantly decreased at 60 dpi under the overexpression of ND1, and may be associated with its therapeutic effect. Additionally, we observed the vessels and did not show any vascular proliferation in the hemorrhagic stroke mouse model (**Additional Figure 3A–I**).

## Discussion

This study showed a significant decrease in brain lesion volume of RIBI mice after *in situ* neuronal regeneration treatment, accompanied by increased neuronal density, alleviated microglial activation, reduced incomplete angiogenesis, and decreased infiltration of CD8^+^ T cells into brain parenchyma, thus ameliorating RIBI. These findings indicate that mechanistically, ND1 *in situ* neural regeneration technology exerts its therapeutic effects by upregulating neurogenesis-related genes and downregulating immune response and angiogenesis-related genes. The effects of *in situ* neuronal regeneration technology in ischemic stroke have been demonstrated previously (Chen et al., 2020; Ge et al., 2020), via converting reactive astrocytes into neurons, but its effect in hemorrhagic stroke has not been determined. Moreover, the process of transdifferentiation in delayed brain injury remains unclear. Our findings showed the conversion of reactive astrocytes into neurons and alleviation of inflammatory response in a hemorrhagic stroke model, indicating a shared therapeutic potential in two different delayed brain injury diseases, both induced by transient insults to the brain. Overall, our study supports the therapeutic potential of *in situ* neuronal regeneration technology for the treatment of delayed brain diseases such as RIBI and hemorrhagic stroke by converting reactive astrocytes into neurons.

In the present study, ND1 treatment decreased lesion volume at 10 weeks compared with that in the RIBI mouse model receiving control virus treatment. Furthermore, we found that ND1 treatment increased neuronal density in the lesion areas of RIBI mice, both by converting reactive astrocytes into neurons and by decreasing the loss of endogenous neurons, which is consistent with the findings by Chen et al. (2020). Upon stimulation of the central nervous system by an insult such as radiation exposure, microglia become activated. Although the phagocytosis of dead cells by activated microglia is vitally important for maintaining the brain microenvironment homeostasis, persistent activation leads to chronic neuroinflammation and aggravates RIBI (Fu et al., 2014; Liu et al., 2022). Moreover, human and animal studies have found that BBB disruption plays a critical role in the pathogenesis of RIBI. Radiation induces incomplete angiogenesis, disrupts the BBB, and causes peripheral immune cells to infiltrate the brain, ultimately exacerbating RIBI (Jiang et al., 2014; Ali et al., 2019; Cheng et al., 2023; Shi et al., 2023a; Zhao et al., 2023). In this study, ND1 treatment exhibited an anti-inflammatory effect, alleviating the microglial activation in the injured area. Furthermore, it reduced incomplete angiogenesis and decreased infiltration of CD8^+^ T cells into brain parenchyma. These results suggest *in situ* neuronal regeneration technology improved the microenvironment of the injured area by reducing microglial activation and BBB disruption, thereby decreasing the loss of endogenous neurons.

Previous sequencing results from an *in vitro* study indicated that ND1 *in situ* neuronal regeneration technology converts reactive astrocytes into neurons by increasing the expression of neurogenesis-related genes and decreasing the expression of astroglia-related genes (Ma et al., 2022). In the RIBI mouse model, high-throughput sequencing results showed that *NeuroD1*, *NeuroD4*, *Sprr1a*, *Gal*, *Klk8*, *Rrs13*, *Ltrm1*, *Adam8*, *Nhlh1* and *Piwil4* significantly increased, and that the expression of A1 astrocyte type-related lineage gene *Lcn2* significantly decreased. Reactive astrocytes in injury or disease can be classified as A1 or A2 type based on their gene expression profile, with A1 exhibiting neurotoxic effects and A2 exhibiting neuroprotective effects (Liddelow et al., 2017). A recent study showed that astrocytes may have immune memory functions and play a proinflammatory role in the progression of chronic neurological diseases (Lee et al., 2024). *Lcn2*, often expressed in reactive astrocytes post-injury (Zamanian et al., 2012), was significantly downregulated in RIBI after ND1 treatment. Additionally, previous reports have indicated the role of ND1 in reducing inflammatory responses (Liu et al., 2020; Zhang et al., 2020). Our sequencing results similarly showed that the immune response-related genes *Ighg2* and *Cxcl5* were significantly decreased in RIBI after ND1 treatment. These results suggest ND1 *in situ* neuronal regeneration technology potentially curbs the inflammatory response of the microenvironment by decreasing the A1 astrocyte type-related genes and immune response-related genes. Moreover, our sequencing results showed downregulation of angiogenesis-related genes (*Esm1* and *Angpt2*) and biological processes in the RIBI after ND1 treatment. Incomplete angiogenesis is one of the typical pathological features of RIBI, which does not form a BBB, leading to leakage of vascular contents and the formation of edema around the lesion (Ali et al., 2019). Therefore, bevacizumab, an anti-angiogenic agent targeting vascular endothelial growth factor, has been effective in RIBI by reducing angiogenesis and cerebral edema (Jiang et al., 2014; Xu et al., 2018; Zhuang et al., 2019; Cai et al., 2020; Garcia et al., 2020). These results suggest that the neuroprotective effect of ND1 overexpression is closely related to its anti-inflammatory and anti-angiogenesis effects.

In addition to the RIBI mice, we investigated the process of transdifferentiation and treatment effects induced by ND1 overexpression in a hemorrhagic stroke mouse model. The findings showed similar transdifferentiation levels at 60 days after virus injection and comparable therapeutic effects in both delayed brain injury mouse models.

A previous study by Wang et al. (2021a) reported neuronal leakage when a high titer AAV (≥ 1E+13 GC/mL) was injected into healthy mouse brains. However, Xu et al. (2022) reported that a high titer can cause severe neuronal leakage and Guo et al. (2023) showed that an excessively high virus titer can lead to tissue damage. In addition, the leakage and glia conversion are two independent events (Chen, 2024). Xiang et al. (2024) used long-term two-photon imaging to continuously capture the process of astrocyte-to-neuron transdifferentiation in lineage-traced mice. Through two-photon calcium imaging and patch clamp recordings, they confirmed that the newly generated neurons established functional connections in local neural circuits, providing the most insightful evidence of astrocyte-to-neuron transdifferentiation. He et al. (2025) found that using different transcription factors (*ND1*, *Ascl1*, *Dlx2*) could transdifferentiate astrocytes into different subtypes of neurons, which cannot be explained by leakage. The virus titer used in this study was 5E+11 GC/mL, which is a working titer that balances high specificity and low inflammatory damage. Together, these previous studies support that the results found in the present study are due to the tissue repair effects of ND1 overexpression rather than leakage.

The present study had some limitations. In the hemorrhagic stroke mouse model, the number of mice was small. Because of the high mortality rate of hemorrhagic stroke models, a mild injury model was used in this study. As a result, there were no changes in the behavioral phenotypes of the injured mice caused by the pathological damage, and transdifferentiation did not alter the behavioral phenotypes of the injured mice. In addition, we did not examine the electrophysiological properties of converted neurons or whether they integrated into the native neural circuits. Additionally, the exact mechanisms underlying the therapeutic effects of ND1 overexpression still need further exploration. We will investigate these questions in future experiments. Like other new technology, *in situ* neural regeneration technology has its own limitations. This technique relies on the transdifferentiation of endogenous reactive astrocytes into neurons within the brain injury area. Therefore, the number of reactive astrocytes in the injury area will affect the therapeutic effect.

In conclusion, our study indicates that *in situ* ND1 overexpression converts reactive astrocytes into neurons in two delayed brain injury mouse models: RIBI and hemorrhagic stroke. In the RIBI mouse model, ND1 overexpression reduced the inflammatory response, incomplete angiogenesis, and the infiltration of peripheral CD8^+^ T cells into the brain parenchyma, thereby significantly reducing the lesion volume. Mechanistically, ND1 *in situ* neural regeneration technology exerted its therapeutic effects by upregulating neurogenesis-related genes and downregulating immune response and angiogenesis-related genes. Overall, our study shows that ND1 *in situ* neural regeneration technology is a potential therapeutic approach for treating delayed brain injuries such as RIBI and hemorrhagic stroke.

## Additional files:

***Additional Figure 1:*** *ND1-treated mice exhibit reduced inflammation and similar vessel characteristics to control mice in brim areas.*

Additional Figure 1 ND1-treated mice exhibited reduced inflammation and similar vessel characteristics to control mice in brim areas.
(A) Representative images of reactive astrocyte-occupied areas (GFAP^+^, green, Alexa Fluor ® 647) after injection with control AAV (left) or ND1 AAV (right). The control virus group had a larger astrocyte-occupied area than the ND1-treated group. Scale bar: 1000 μm. (B) Quantitative analysis of GFAP positive areas (*n* = 5). (C) Representative images of GFAP^+^ (green, Alexa Fluor ® 647) core and brim areas. Scale bar: 1000 μm. (D) Confocal images (63× magnification) of Iba1 (green, microglial marker, Alexa Fluor ® 488) and CD68 (red, activated microglial marker, Alexa Fluor ® 647). The control virus group had a higher mean intensity of Iba1 and CD68 than the ND1-treated group in brim areas. Scale bar: 20 μm. (E, G) Quantitative analysis of Iba1 mean intensity in core and brim areas (*n* = 6). (F, H) Quantitative analysis of CD68 mean intensity in core and brim areas (*n* = 6). (I) Confocal images (40× magnification) of CD31 (green, vascular endothelial cell marker, Alexa Fluor ® 488) and AQP4 (red, astrocytic endfoot marker, Alexa Fluor ® 647) under different conditions. There was no significant difference of vessels between the two groups in brim areas. Scale bar: 20 μm. (J, K) Quantitative analysis of total vessel length and vessel density in brim areas (*n* = 6). Data are expressed as mean ± SD. *P < 0.05, **P < 0.01, ***P < 0.001, ****P < 0.0001 (unpaired Student's *t*-test). AAV: Adeno-associated virus; AQP4: aquaporin-4; GFAP: glial fibrillary acidic protein; Iba1: ionized calcium binding adapter molecule 1; ND1: NeuroD1; RA: radiation; RA+ND1: radiation + NeuroD1; RA+Ctrl virus: radiation + control virus.

***Additional Figure 2:*** *The protein interaction networks for RN vs. RC.*

Additional Figure 2 The protein interaction networks for RN *vs*. RC.
The purple circles represent the protein NeuroD1, which holds core positions in the networks. Upregulated genes were represented as pink circles, and downregulated genes were represented as blue diamonds. Genes associated with cell differentiation, immune response, and tubular structure development were highlighted with red, green, and blue circles, respectively (*n* = 3). RC: radiation + control virus; RN: radiation + NeuroD1 virus.

***Additional Figure 3:*** *There is no significant difference of vessels between control and ND1 virus treatment groups in hemorrhagic stroke mouse brain.*

Additional Figure 3 There is no significant difference of vessels between control and ND1 virus treatment groups in hemorrhagic stroke mouse brain.
(A-C) Confocal images (63× magnification) of CD31 (green, vascular endothelial cell marker, Alexa Fluor ® 488) and AQP4 (red, astrocytic endfoot marker, Alexa Fluor ® 647) under different conditions at 7, 30 and 60 dpi. There was no significant difference of vessels between the two groups. Scale bar: 20 μm. (D-F) Quantitative analysis showing no significant difference of the vessel density between the two groups at 7, 30 and 60 dpi. (G-I) Quantitative analysis showing no significant difference of total vessel length between the two groups at 7, 30 and 60 dpi. Data are expressed as mean ± SD (*n* = 3) and were analyzed by unpaired Student's t-test. AQP4: aquaporin-4; Ctrl: control; dpi: days post-virus injection; ND1: NeuroD1; ns: not significant.

## Data Availability

*All data generated or analyzed during this study are included in the manuscript and its Additional files*.
