# Supplementary figures and images for: NeuroD1-based in situ neural regeneration for the treatment of radiation-induced brain injury
Source: Neural Regen Res. 2025 Jan 29;21(7):3035–45. doi: 10.4103/NRR.NRR-D-24-01067 (PMC13378961; doi:10.4103/NRR.NRR-D-24-01067)

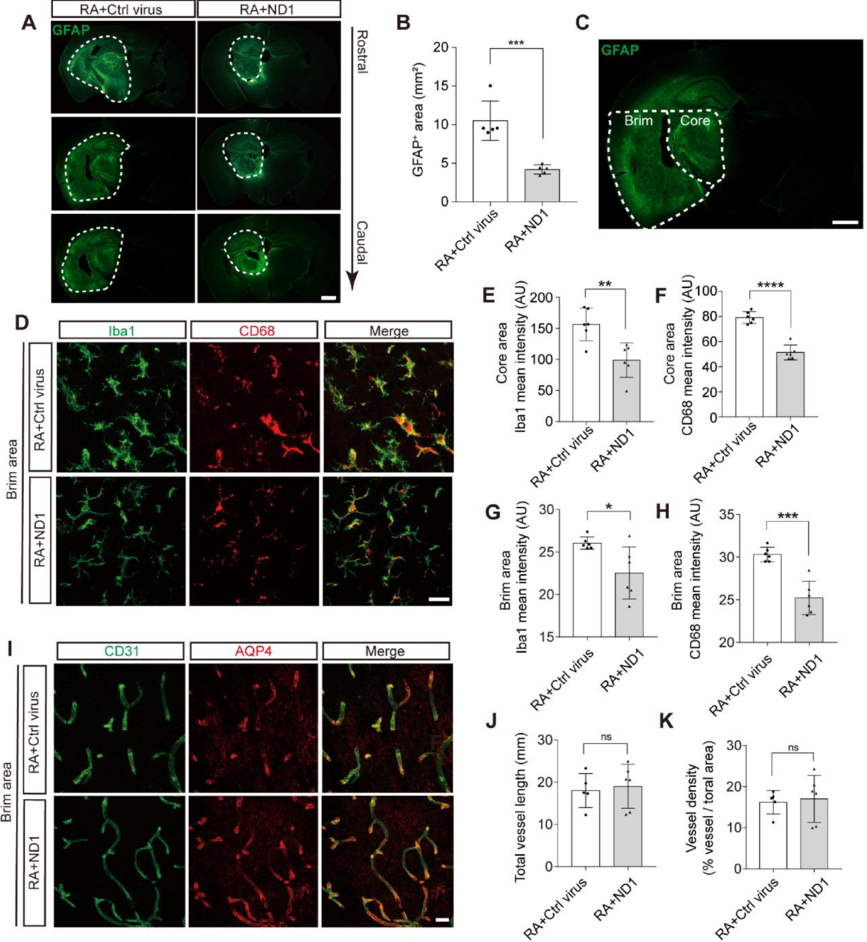

Supplement: Supplementary file 1 [file NRR-21-3035_Suppl1.tif]

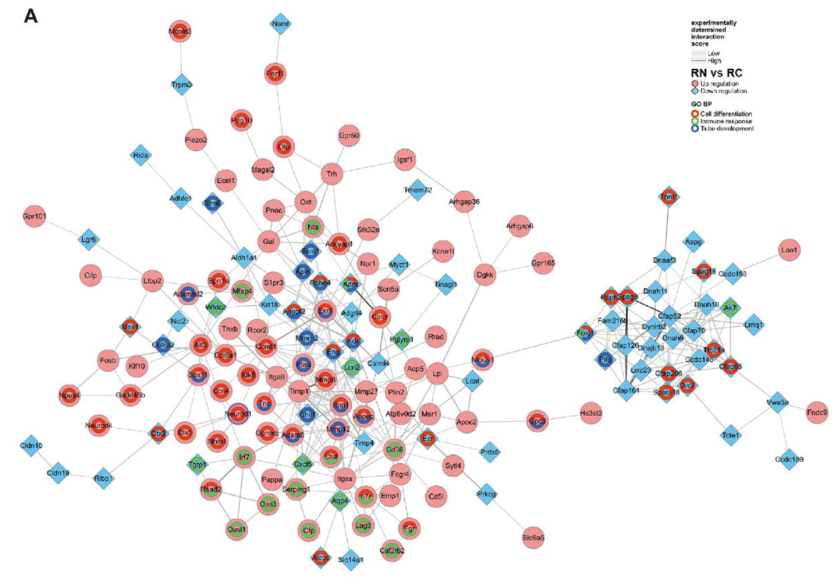

Supplement: Supplementary file 2 [file NRR-21-3035_Suppl2.tif]

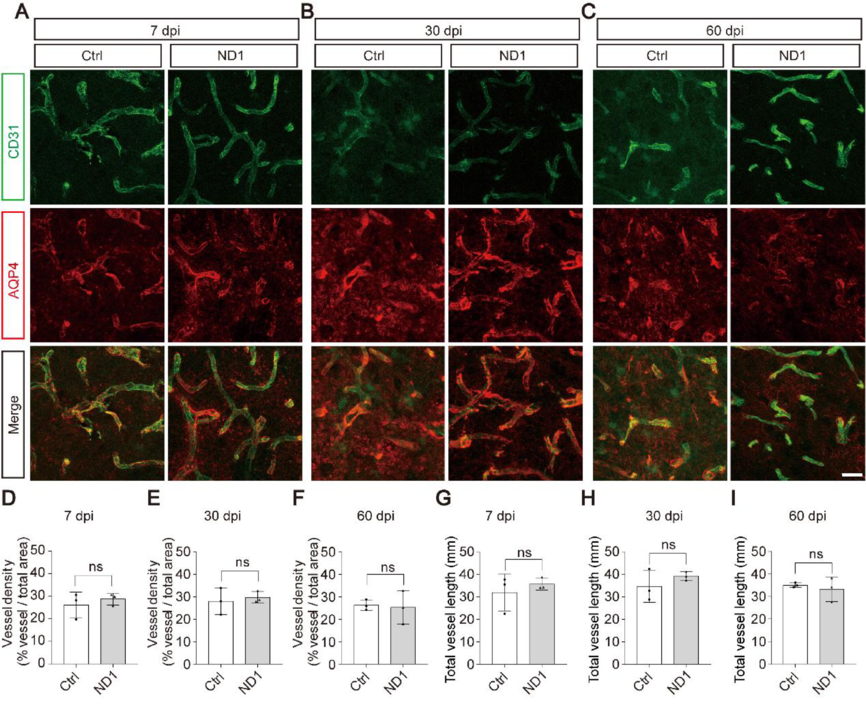

Supplement: Supplementary file 3 [file NRR-21-3035_Suppl3.tif]
